# Magnitude of Visual Acuity Change with ETDRS versus Snellen Testing in Clinical Trials: Implications for Clinic-Based Outcomes

**DOI:** 10.1016/j.xops.2023.100372

**Published:** 2023-07-19

**Authors:** Mirataollah Salabati, Charles Huang, Alireza Kamalipour, Hannah J. Yu, Raziyeh Mahmoudzadeh, Karen Jeng-Miller, Eric Chen, Chirag P. Shah, Charles C. Wykoff, Jason Hsu

**Affiliations:** 1 The Retina Service of Wills Eye Hospital, Mid Atlantic Retina, Philadelphia, Pennsylvania; 2 Hamilton Glaucoma Center, Shiley Eye Institute, Viterabi Family Department of Ophthalmology, UC San Diego, La Jolla, California; 3 Retina Consultants of Texas, Retina Consultants of America, Houston, Texas; 4 Ophthalmic Consultants of Boston, Boston, Massachusetts

**Keywords:** Clinical trial, ETDRS, Snellen, Visual acuity change

## Abstract

**Purpose:**

To compare visual acuity (VA) changes using standardized ETDRS best-corrected visual acuity (BCVA) and nonstandardized Snellen VA among subjects enrolled in clinical trials.

**Design:**

Retrospective study.

**Participants:**

Patients enrolled in prospective clinical trials at 3 urban retina practices.

**Methods:**

Best available Snellen VA at the clinic visit before study entry and after exit were compared with the ETDRS BCVA at trial entry and exit. The correlation and discrepancies between Snellen VA and ETDRS methods as well as the VA changes from trial entry to exit were evaluated.

**Main Outcome Measures:**

The discrepancy between VA change from trial entry to exit using Snellen VA versus ETDRS BCVA methods.

**Results:**

A total of 273 eyes were included. The mean (standard deviation [SD]) Snellen VA was 58.1 (20) ETDRS-equivalent letters (Snellen 20/69) at the clinic visit before trial entry and 61.6 (21) ETDRS-equivalent letters (Snellen 20/59) at the visit after trial exit. The mean (SD) ETDRS BCVA was 65.5 (16) letters (Snellen 20/49) at trial entry and 70.5 (17) letters (Snellen 20/39) at trial exit. The mean VA change from trial entry to exit was not significantly different for ETDRS (5 letters of vision gain) compared with Snellen (3.6 letters of vision gain) methods (*P* = 0.061). Eyes with baseline Snellen VA 20/50 or worse gained significantly more letters using Snellen (9.3 ± 22.3 letters) compared with ETDRS (5.2 ± 18.7 letters; *P* = 0.012). Among eyes with baseline Snellen VA of > 20/50, VA gain was significantly greater with the ETDRS method (4.9 ± 12.3 letters) compared with Snellen (−1.5 ± 12.3 letters; *P* < 0.001).

**Conclusions:**

The mean VA change from clinical trial entry to exit was similar between the ETDRS and Snellen methods. However, among patients with worse baseline Snellen vision, the magnitude of VA change was greater with Snellen compared with ETDRS, whereas among those with better baseline vision, this magnitude was greater with the ETDRS method. Understanding the proportion of the study population with varying VA levels may have implications for interpreting VA outcomes from retrospective clinic-based studies compared with those reported in clinical trials.

**Financial Disclosure(s):**

Proprietary or commercial disclosure may be found after the references.

The most common method for measuring visual acuity (VA) in clinical practice across the United States is the Snellen Chart. This method was first introduced by Dr Hermann Snellen in 1862^1^ and consists of lines with letters decreasing in size from top to bottom. The numerator is the distance from the chart, and the denominator is the distance at which a letter corresponds to the visual angle of 5 minutes of arc. However, in the clinical trial setting, the US Food and Drug Administration only accepts standardized protocol refraction using ETDRS charts for trial registrations and also for the visual outcomes.^1^

Multiple retrospective clinic-based studies have suggested that patients with exudative retinal diseases often have worse visual outcomes compared with those achieved in pivotal clinical trials.^2^, ^3^, ^4^, ^5^, ^6^ Several reasons for this disparity have been proposed. Most retrospective studies include patients who would not have met clinical trial eligibility requirements. In addition, patients in these clinic-based studies seem to receive fewer injections and/or have poor treatment adherence. Another possibility that has not been well explored is the effect of differences in VA measurement. The retrospective studies often convert the Snellen VA values to ETDRS letters using suggested formulas to compare them with the results of clinical trials. In addition, clinical trials typically use standardized VA testing and refraction protocols for theoretically obtaining a more accurate and reproducible best-corrected VA (BCVA), whereas the clinic-based studies rely on habitual correction and/or pinhole VA using nonstandardized measuring environments. Although the ETDRS BCVA and Snellen VA approaches have been shown to be highly correlated, multiple studies have demonstrated that ETDRS tends to produce significantly better VA scores in comparison to Snellen VA.^1^^,^^7^, ^8^, ^9^ Therefore, when comparing clinical trial outcomes with retrospective clinic-based outcomes, it is essential to consider the discrepancies between these 2 methods. To better understand this discrepancy, the current study compared VA change using nonstandardized Snellen VA with standardized ETDRS BCVA among patients enrolled in prospective clinical trials.

## Methods

This was a retrospective multicenter study of participants enrolled in prospective clinical trials at the Retina Service of Wills Eye Hospital and offices of Mid Atlantic Retina (Philadelphia, Pennsylvania), Retina Consultants of Texas (Houston, Texas), and Ophthalmic Consultants of Boston (Boston, Massachusetts). Institutional review board approval was obtained from each study site, encompassing a thorough review of the study’s data collection and statistical analysis. The research adhered to the tenets of the Declaration of Helsinki and was conducted in compliance with Health Insurance Portability and Accountability Act regulations. Given the retrospective nature of this study, the need for informed consent was waived by the institutional review board. The titles, sponsors, and identifiers of the clinical trials are provided in the supplementary material.

Demographic data including age, sex, race/ethnicity, primary diagnosis, and lens status were collected. For each subject, the dates of the study entry and exit as well as the last clinic visit before study entry and the first clinic visit after the study exit were determined. The data collection period on VA measurements spanned from January 2006 (the earliest trial) to March 2021 (the latest trial). The Snellen VA at the last visit before entering the study and the first visit after exiting the study and ETDRS BCVA at trial initiation and conclusion were collected. The difference between the 2 Snellen measurements was calculated and labeled as Snellen change. Similarly, the difference between the 2 ETDRS measurements was labeled ETDRS change. The Snellen VAs were performed by ophthalmic technicians in nonstandardized clinic examination lanes. In cases where multiple VAs were reported (i.e., without correction, with correction, and pinhole), the best available VA was selected. ETDRS BCVA measurements were performed using standardized refraction protocols by certified examiners in certified examination lanes. Patients with a > 90-day interval between Snellen and ETDRS measurements before and/or after the clinical trial were excluded from the final analysis.

The statistical analysis was performed using SPSS software (version 24; IBM Corporation). The Snellen VA was converted to ETDRS-equivalent letters using the following formula:

ETDRS−equivalentletters=85+50×log(SnellenFraction)

Snellen fraction was obtained for patients with counting fingers (CFs) vision as follows: CF 1 foot = 1/200; CF 2 feet = 2/200; CF 4 feet = 4/200, etc.^10^ Based on a paper by Gregori et al,^11^ the following letter scores were used: 1/200 (0 letters), 2/200 (2 letters), 3/200 (4 letters), 4/200 (6 letters), 5/200 (8 letters), and 6/200 (10 letters).

Spearman’s correlation coefficient was utilized to examine the strength of the correlation between the ETDRS and Snellen methods. Wilcoxon signed-rank test was performed to evaluate the difference between Snellen and ETDRS values at trial entry and exit as well as the difference in the magnitude of VA change from trial entry to exit using the 2 methods. A linear regression model was used to examine the association between Snellen VA level and the discrepancy between Snellen VA and ETDRS BCVA. A 2-sided *P* value of < 0.05 was considered statistically significant.

## Results

A total of 273 eyes of 273 patients were included in the analysis. The mean (standard deviation [SD]) age at study entry was 68.7 (13.6) years (range, 25–93). The right eye was the study eye in 131 (48%) of the participants, and 178 (48.1%) were female. Among the patients who disclosed their race/ethnicity, 195 (71.4%) were White. Enrollment in the clinical trials was for neovascular age-related macular degeneration (nAMD) in 166 (60.8%) eyes, nonproliferative diabetic retinopathy without diabetic macular edema (DME) in 12 (4.4%) eyes, nonproliferative diabetic retinopathy with DME in 34 (12.5%) eyes, proliferative diabetic retinopathy in 36 (13.2%) eyes, and retinal vein occlusion in 25 (9.2%) eyes. The median (interquartile range) number of days between Snellen and ETDRS measurements before study entry was 14 (7–33) days and after the study exit and return to the clinic was 30 (28–42) days.

### Correlation between ETDRS BCVA and Snellen VA before Study Entry

The mean (SD) Snellen VA at the clinic visit before study entry was 58.1 (20) ETDRS-equivalent letters (Snellen 20/69). The mean (SD) ETDRS BCVA at study entry was 65.5 (16) letters (Snellen 20/49). There was a significant correlation between Snellen VA and ETDRS BCVA (*r* = 0.725, *P* < 0.001). The ETDRS method indicated an average of 7.5 letters (95% CI, 5.8–9.2) better VA compared with the Snellen method (*P* < 0.001). The ETDRS method indicated better average VA values in both phakic (6.5 letters; n = 150; *P* < 0.001) and pseudophakic eyes (8.7 letters; n = 123; *P* < 0.001) compared with the Snellen method, and the lens status was not associated with the discrepancy between the 2 methods (*P* = 0.21).

### Correlation between ETDRS BCVA and Snellen VA after Study Exit

The mean (SD) ETDRS BCVA at study exit was 70.5 (17) letters (Snellen 20/39), and the mean (SD) Snellen VA at the first clinic visit after study exit was 61.6 (21) ETDRS-equivalent letters (Snellen 20/59). There was a significant correlation between the last ETDRS BCVA at study exit and the first clinic Snellen VA after study exit (*r* = 0.822, *P* < 0.001). The ETDRS method indicated an average of 8.9 letters (95% CI, 7.6–10.3) better VA than the Snellen method (*P* < 0.001). The mean ETDRS BCVA was better compared with the mean Snellen VA in both phakic (8.1 letters; n = 131; *P* < 0.001) and pseudophakic eyes (9.6 letters; n = 142l *P* < 0.001), and the lens status was not associated with the discrepancy between the 2 methods (*P* = 0.27). Table 1 shows the discrepancy of ETDRS BCVA and Snellen VA measurement methods classified based on the different retinal pathologies before entering and after exiting the study.

**Table 1:** The Discrepancy of ETDRS and Snellen Method at Study Entry and Exit Stratified by Retinal Pathology

Disease Category	N	Mean ETDRS Letters at Study Entry (Snellen Equivalent)	Mean Snellen VA Converted to ETDRS Letters at Clinic Visit before Study Entry (Snellen Equivalent)	Mean Letter Difference	*P*	Mean ETDRS Letters at Study Exit (Snellen Equivalent)	Mean Snellen VA Converted to ETDRS Letters on Return to Clinic (Snellen Equivalent)	Mean Letter Difference	*P*
nAMD	166	65 (20/50)	55 (20/80)	10	< 0.001	68.5 (20/43)	57.5 (20/71)	11	< 0.001
NPDR without DME	12	85.5 (20/19)	78.5 (20/27)	7	0.01	87 (20/18)	78.5 (20/27)	8.5	0.002
NPDR with DME	34	62.5 (20/56)	56.5 (20/74)	6	0.03	71.5 (20/37)	63.5 (20/54)	8	< 0.001
PDR	36	76.5 (20/29)	71.5 (20/37)	5	< 0.001	82.5 (20/22)	74.5 (20/32)	8	< 0.001
RVO	25	47.5 (20/112)	43 (20/138)	4.5	0.20	60 (20/63)	48 (20/110)	12	0.002

DME = diabetic macular edema; nAMD = neovascular age-related macular degeneration; NPDR = non-proliferative diabetic retinopathy; PDR = proliferative diabetic retinopathy; RVO = retinal vein occlusion; VA = visual acuity.

### Analysis Based on VA Level

**Figure 1:**
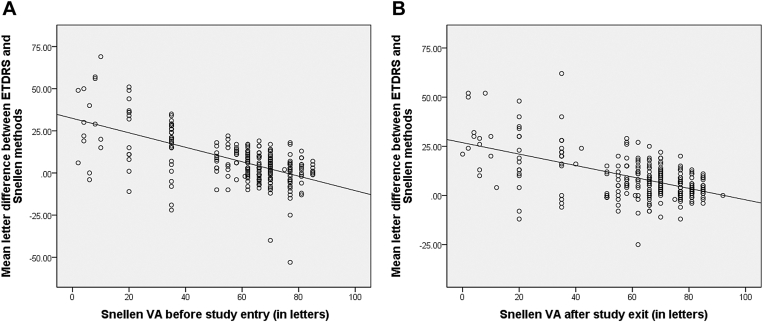
The letter difference between Snellen and ETDRS methods based on the baseline Snellen visual acuity (VA) **(A**) before study entry and **(B**) after trial exit. The difference between ETDRS and Snellen measurements increases with worsening of baseline Snellen VA.

**Figure 2:**
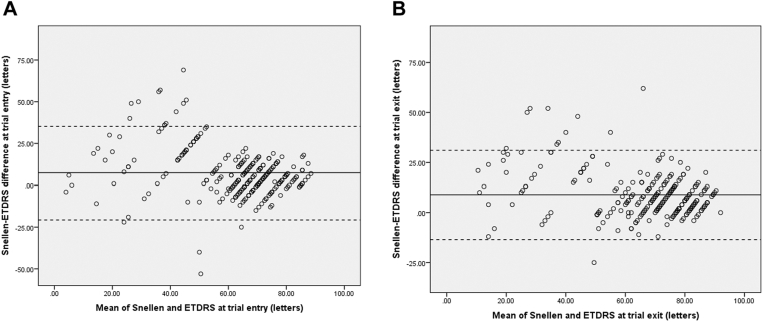
Bland-Altman analysis of Snellen and ETDRS variability (**A**) before trial entry and (**B**) after trial exit. The x axis represents the average of the Snellen and ETDRS letter scores, and the y axis represents the difference between the Snellen and ETDRS scores for each eye. The solid line is the mean difference between Snellen and ETDRS. The dotted lines are the 95% limits of agreement.

A linear regression analysis was used to evaluate the effect of Snellen VA level on the discrepancy between the Snellen and ETDRS methods. There was a statistically significant correlation between the level of VA and the discrepancy between the 2 methods both before trial entry and after trial completion (*P* < 0.001, respectively). The worse the Snellen VA, the greater the discrepancy between ETDRS BCVA and Snellen VA (Fig 1). Using Bland-Altman analysis, the difference between Snellen and ETDRS was plotted against their means at trial entry and exit, showing a higher discrepancy between Snellen and ETDRS at lower VAs compared with better VAs (Fig 2). Eyes at study entry with Snellen VA of CF (n = 15) had a mean (SD) discrepancy of 30.5 (22) letters, whereas, among eyes with Snellen VA better than CF (n = 258), the mean discrepancy was 6.1 (12) letters (*P* < 0.001). Similarly, in eyes exiting the trial, the mean discrepancy (SD) was 28 (15) letters for those with Snellen VA of CF (n = 14) after return to clinic and 7.8 (10) letters for those with Snellen VA better than CF (n = 259; *P* < 0.001).

### Analysis of ETDRS BCVA Change and Snellen VA Change

There was a moderate correlation between the ETDRS BCVA change (5.0 ± 14 letters) and Snellen VA change (3.6 ± 18.6 letters) from study entry to exit (*r* = 0.50; *P* < 0.001). No significant difference was found between the average VA change for the EDTRS (5 letters of vision gain) and the Snellen (3.6 letters of vision gain; *P* = 0.061) methods. However, when comparing the ETDRS versus Snellen change above or below certain baseline VA cutoffs, eyes with better baseline Snellen VA gained more letters by the EDTRS method, whereas eyes with worse baseline Snellen VA gained more letters by the Snellen method. For example, eyes with baseline Snellen VA of 20/50 or worse gained significantly more letters using Snellen (9.3 letters) compared with ETDRS (5.2 letters; *P* = 0.012). However, among eyes with baseline Snellen VA better than 20/50, VA gain was significantly greater with the ETDRS method (4.9 letters) than Snellen (−1.5 letters; *P* < 0.001). Table 2 shows the comparisons at different VA cutoffs.

**Table 2:** The Comparison between ETDRS and Snellen Methods for the Magnitude of Vision Change from Trial Entry to Exit Based on Different Baseline Snellen VA Cutoffs

VA Cutoff	N	ETDRS (Letters)	Snellen (Letters)	*P*
20/200				
≤ 20/200	64	7.4	17.7	< 0.001
> 20/200	209	4.3	−.76	< 0.001
20/60				
≤ 20/60	96	4.7	11	0.002
> 20/60	177	5.1	−0.45	< 0.001
20/50				
≤ 20/50	130	5.2	9.3	0.012
> 20/50	143	4.9	−1.5	< 0.001

VA = visual acuity.

**Figure 3:**
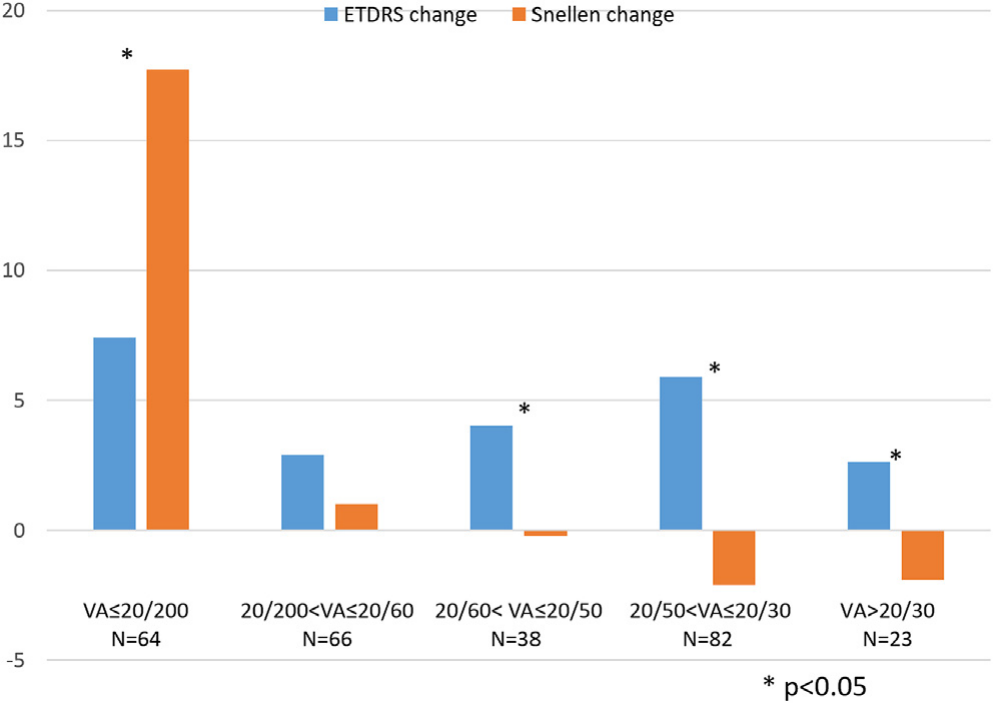
ETDRS and Snellen changes based on baseline Snellen visual acuity (VA) quintiles. The Snellen method shows significantly higher letter gains compared with ETDRS in subjects with worse baseline VA. Conversely, the ETDRS method shows significantly higher letter gains compared with Snellen in subjects with better baseline VA.

A separate analysis was performed by dividing the data set into quintiles. On average, eyes with baseline Snellen VA of 20/200 or worse gained 10 (95% CI, 4.5–16.1) more letters with the Snellen method compared with ETDRS. However, the ETDRS method demonstrated 1.9 (95% CI, −1.1 to 4.9) more letters gained in eyes with 20/200 < VA ≤ 20/60, 4.3 (95% CI, 0.55–7.9) more letters in eyes with 20/60 < VA ≤ 20/50, 8.0 (95% CI, 4.8–11.2) more letters in eyes with 20/50 < VA ≤ 20/30, and 4.6 (95% CI, 1.6–7.5) more letters in eyes with > 20/30 compared with the Snellen method. Figure 3 summarizes the ETDRS versus Snellen change in different quintiles.

## Discussion

In the current study, we evaluated the discrepancy between ETDRS and Snellen VA measurements before entering and after exiting clinical trials as well as the discrepancy of VA changes between ETDRS and Snellen methods from clinical trial entry to exit. Although our findings did not indicate a significant difference between average EDTRS versus Snellen VA changes, we demonstrated that the magnitude of EDTRS versus Snellen VA change varies depending on the baseline Snellen VA of the participants. Therefore, the magnitude of VA change obtained by either of these methods should be interpreted according to patients’ baseline VA.

Multiple retrospective studies analyzing patients in routine clinical practice have reported inferior visual outcomes compared with those achieved in clinical trials. For example, an analysis of 49 485 eyes with nAMD from the Vestrum Health Retina Database found that, after a mean of 7.3 anti-VEGF injections, at 1 year, the mean vision gained was 1 ETDRS letter.^2^ Similarly, a study evaluating a database of 13 859 patients with nAMD from the Intelligent Research in Sight registry reported that at 1 year and after receiving a mean of 6.1 anti-VEGF injections, mean vision improvement was 2.5 letters.^12^ In comparison, the registration trials for ranibizumab and aflibercept in nAMD have shown 8.5 ETDRS letters vision gain after 1 year of treatment and 7 to 12 anti-VEGF injections.^13^, ^14^, ^15^ A similar discrepancy has been identified among patients receiving anti-VEGF injections for DME. A study by Ciulla et al^3^ involving 15 608 eyes with DME from the Vestrum Health Retina Database reported a vision gain of 5.5, 5.5, and 4.0 letters in aflibercept, bevacizumab, and ranibizumab-treated patients, respectively, at 1 year. In contrast, 1-year results from Diabetic Retinopathy Clinical Research protocol T demonstrated 13.3, 9.7, and 11.2 letters of vision improvement among aflibercept-, bevacizumab-, and ranibizumab-treated patients respectively.^16^ Apart from undertreatment and less rigorous follow-up in outpatient clinical practice, one potential reason for this inferiority could be discrepant methods of VA assessment utilized in clinical trials versus routine clinical practice.

When evaluating patients with baseline Snellen VA of ≤ 20/50, the Snellen method yielded higher levels of VA change compared with ETDRS by around 4 letters. On the other hand, when baseline Snellen VA was > 20/50, the ETDRS method produced higher levels of VA change compared with the Snellen by around 6.5 letters. Even greater discrepancies were seen when comparing the Snellen and ETDRS methods among patients with baseline Snellen VA thresholds of 20/60 and 20/200. With ETDRS giving much higher scores compared with Snellen among patients with worse VA before and after trial participation, it is plausible to expect less change in ETDRS from entry to exit compared with Snellen in patients with poor baseline VA. Because retrospective studies using clinic-based data often include patients with lower Snellen VA than clinical trial inclusion criteria, one might expect to see larger Snellen VA changes in the real-world, depending on the proportion of such patients included in a given study. On the other hand, retrospective studies that restrict patients to similar Snellen VA inclusion criteria as the ETDRS BCVA used in clinical trials or have a high proportion of such patients might underestimate the magnitude of VA change. In the current study, the ETDRS method produced higher levels of VA change compared with Snellen by around 5 letters when baseline Snellen VA was better than 20/200. One hypothetical example may be derived using the previously mentioned Intelligent Research in Sight study in nAMD.^12^ Assuming the real-world Snellen VA in that study followed a similar distribution to our study but included only patients with 20/200 or better baseline vision, the actual VA change may have been approximately 7.5 letters, instead of the reported 2.5 letters had an ETDRS method been used, which would then suggest real-world outcomes are not as poor as we have believed. Overall, these findings highlight the importance of understanding the breakdown by baseline Snellen VA of patients included in real-world analyses to appreciate the potential influence on VA change when compared with clinical trials.

Compared with the Snellen method, ETDRS BCVA values were 7.5 and 8.9 letters greater at study entry and exit, respectively (*P* < 0.001). More than 70% of the eyes at study entry and 80% of the eyes at study exit had better scores on ETDRS compared with Snellen. This discrepancy seemed to be independent of lens status and baseline retinal pathology. Looking at the effect of baseline VA level, regression analysis demonstrated that the discrepancy between the 2 methods increased significantly with worse Snellen VA. These results are in agreement with previous studies that have evaluated the discrepancy of the Snellen and ETDRS methods in either a routine retina practice^1^^,^^8^ or among subjects entering prospective retina trails.^9^ Specifically, VA tends to be 6.1 to 7.5 letters (approximately 1–2 lines) higher with ETDRS compared with the Snellen method, and our results are in line with what has been published before. In our study, we also demonstrated that the discrepancy continues to exist after trial conclusion. However, unlike previous studies that looked at the discrepancies at one specific time point, our study evaluated the discrepancies from clinical trial entry to exit and reported the discrepancies between the magnitude of VA change comparing the 2 methods, which has not been reported before.

There are multiple reasons for such discrepancies. Snellen Charts are not standardized and uniform among all manufacturers, and, as mentioned before, the number of letters is not consistent among all vision lines. Therefore, missing the same number of letters on different lines does not have a similar impact on Snellen VA, whereas ETDRS charts contain the same number of letters on each line and have an equal logarithmic decrement in the size of letters between each successive line, making the results more reliable and reproducible. The ETDRS measurements are performed according to a prespecified protocol refraction guideline in a clinical trial setting and follow a standardized pattern among different involved sites, whereas no such protocols are performed for Snellen VA testing, likely leading to greater test–retest variability among examiners and different sites when utilizing Snellen assessment.

This study has some limitations associated with its retrospective nature. The Snellen VA measurements were performed in a nonstandardized fashion without a protocol refraction, whereas the ETDRS BCVA measurements were standardized. Although this may contribute to greater variations and inaccuracy in Snellen VA readings, it reflects typical outpatient clinical settings, which was the aim of this study. The Snellen and ETDRS measurements were performed within a median interval of 14 days (before trial initiation) and 30 days (after trial conclusion). With an increasing interval between 2 measurements, there may be a higher chance of fluctuation in retinal pathology and, therefore, increasing discrepancies in VA. Another limitation is that we included clinical trials enrolling patients with different retinal diseases. It is possible that discrepancies in Snellen VA and ETDRS BCVA may vary by disease state.

In conclusion, among patients with better baseline Snellen vision, the magnitude of VA change was significantly greater with the ETDRS method compared with Snellen. Conversely, among patients with worse baseline Snellen vision, the magnitude of VA change was greater with Snellen compared with ETDRS. These findings may have implications for interpreting VA outcomes in retrospective clinic-based studies compared with clinical trials. In particular, the proportion of patients included in retrospective studies with better versus worse Snellen vision at baseline should be analyzed because this metric may influence the reported magnitude of VA change.
