# Supplementary material for: Magnitude of Visual Acuity Change with ETDRS versus Snellen Testing in Clinical Trials: Implications for Clinic-Based Outcomes
Source: Ophthalmol Sci. 2023 Jul 19;4(2):100372. doi: 10.1016/j.xops.2023.100372 (PMC10587620; doi:10.1016/j.xops.2023.100372)
Supplement: Supplementary Data [file mmc1.pdf]

Trials that had at least 5 patients eligible for this study were as follows:

1. Superdose Anti-VEGF (SAVE, Genentech, South San Francisco, CA) [*Brown DM, Chen E, Mariani A, Major JC, SAVE Study Group. Super-dose anti-VEGF (SAVE) trial: 2.0 mg intravitreal ranibizumab for recalcitrant neovascular macular degeneration-primary end point. Ophthalmology. 2013;120(2):349-354. doi:10.1016/j.ophtha.2012.08.008*]

2. Intravitreal Aflibercept for Retinal Nonperfusion in Proliferative Diabetic Retinopathy (RECOVERY, Regeneron, Tarrytown, NY, ClinicalTrials.gov identifier: NCT02863354) [*Wykoff CC, Nittala MG, Zhou B, et al. Intravitreal Aflibercept for Retinal Nonperfusion in Proliferative Diabetic Retinopathy: Outcomes from the Randomized RECOVERY Trial. Ophthalmol Retina. 2019;3(12):1076-1086. doi:10.1016/j.oret.2019.07.011*]

3. A Safety and Efficacy Study of Abicipar Pegol in Participants with Neovascular Age-related Macular Degeneration (CEDAR, Allergan, Dublin, Ireland, ClinicalTrials.gov Identifier: NCT02462928) [Kunimoto D, Yoon YH, Wykoff CC, et al. Efficacy and Safety of Abicipar in Neovascular Age-Related Macular Degeneration: 52-Week Results of Phase 3 Randomized Controlled Study. *Ophthalmology*. 2020;127(10):1331-1344. doi:10.1016/j.ophtha.2020.03.035]

4. Aflibercept for Subjects with Exudative AMD who were Incomplete Responders to Multiple Ranibizumab Anti-VEGF Injections (TURF, Regeneron, ClinicalTrials.gov Identifier NCT 01543568) [*Wykoff CC, Brown DM, Maldonado ME, Croft DE. Aflibercept treatment for patients with exudative age-related macular degeneration who were incomplete responders to multiple ranibizumab injections (TURF trial). Br J Ophthalmol. 2014;98(7):951-955. doi:10.1136/bjophthalmol-2013-304736]*

5. Treat-and-Extend Age-Related Macular Degeneration (TREX-AMD, Genentech, ClinicalTrials.gov Identifier: NCT01748292) [*Wykoff CC, Ou WC, Croft DE, et al. Neovascular age-related macular degeneration management in the third year: final results from the TREX-AMD randomised trial. Br J Ophthalmol. 2018;102(4):460-464. doi:10.1136/bjophthalmol-2017-310822*]

6. Intravitreal Aflibercept as Indicated by Real-Time Objective Imaging to Achieve Diabetic Retinopathy Improvement (PRIME, Regeneron, ClinicalTrials.gov Identifier: NCT03531294) [*Intravitreal Aflibercept as Indicated by Real-Time Objective Imaging to Achieve Diabetic Retinopathy Improvement - Tabular View - ClinicalTrials.gov. Accessed February 15, 2022. https://clinicaltrials.gov/ct2/show/record/NCT03531294*]

7. Efficacy and Safety of RTH258 Versus Aflibercept - Study 1 (HAWK, Alcon, Geneva, Switzerland, ClinicalTrials.gov Identifier: NCT02307682) [*Dugel PU, Koh A, Ogura Y, et al. HAWK and HARRIER: Phase 3, Multicenter, Randomized, Double-Masked Trials of Brolucizumab for Neovascular Age-Related Macular Degeneration. Ophthalmology. 2020;127(1):72-84. doi:10.1016/j.ophtha.2019.04.017].*

8. Wide-Field Angiography Guided Targeted Retinal Photocoagulation Combined with Anti-VEGF Intravitreal Injections for the Treatment of Ischemic Retinal Vein Occlusion (WAVE, Genentech, ClinicalTrials.gov Identifier: NCT01710839), Treat-and-Extend Diabetic Macular Edema (TREX-DME, ClinicalTrials.gov identifier: NCT01934556) [*Payne JF, Wykoff CC, Clark WL, et al. Randomized Trial of Treat and Extend Ranibizumab With and Without Navigated Laser Versus Monthly Dosing for Diabetic Macular Edema: TREX-DME 2-Year Outcomes. Am J Ophthalmol. 2019;202:91-99. doi:10.1016/j.ajo.2019.02.005*]

9. Efficacy and Safety of Intravitreal Injections Combined with Panretinal Photocoagulation for Clinically Significant Macular Edema Secondary to Diabetes Mellitus (DAVE), Safety and Efficacy of Suprachoroidal CLS-TA Alone or in Combination with Intravitreal Aflibercept for the

Treatment for Diabetic Macular Edema (HULK, Clearside Biomedical, Alpharetta, GA, ClinicalTrials.gov identifier: NCT02949024) [*Wykoff CC, Khurana RN, Lampen SIR, et al. Suprachoroidal Triamcinolone Acetonide for Diabetic Macular Edema: The HULK Trial. Ophthalmol Retina. 2018;2(8):874-877. doi:10.1016/j.oret.2018.03.008*]

10. Rubeosis Anti-VEGF (RAVE, ClinicalTrials.gov identifier: NCT00406471) [*Brown DM, Wykoff CC, Wong TP, et al. Ranibizumab in preproliferative (ischemic) central retinal vein occlusion: the rubeosis anti-VEGF (RAVE) trial. Retina Phila Pa. 2014;34(9):1728-1735. doi:10.1097/IAE.0000000000000191*]

11. Lucentis Utilizing Visudyne (LUV, Novartis, Basel, Switzerland, ClinicalTrials.gov Identifier: NCT00473642) [*Chen E, Brown DM, Wong TP, et al. Lucentis using Visudyne study: determining the threshold-dose fluence of verteporfin photodynamic therapy combined with intravitreal ranibizumab for exudative macular degeneration. Clin Ophthalmol Auckl NZ. 2010;4:1073-1079. doi:10.2147/OPTH.S13969*]
